# Selective Expansion of CD34+ Cells from Mouse Bone Marrow Cultured on LH/P MP-Coated Plates with Adequate Cytokines

**DOI:** 10.1177/2041731411425419

**Published:** 2011-10-30

**Authors:** Satoko Kishimoto, Masayuki Ishihara, Yasuhiro Kanatani, Masaki Nambu, Megumi Takikawa, Yuki Sumi, Shingo Nakamura, Yasutaka Mori, Hidemi Hattori, Yoshihiro Tanaka, Toshinori Sato

**Affiliations:** 1 Research Institute, National Defense Medical College, Tokorozawa, Saitama 359-8513, Japan; 2 Research Fellow of the Japan Society for the Promotion of Science, Tokyo 102-8472, Japan; 3 Department of Policy Science, National Institute of Public Health, Wako, Saitama 351-0197, Japan; 4 Department of Plastic and Reconstructive Surgery, National Defense Medical College, Tokorozawa, Saitama 359-8513, Japan; 5 Department of Surgery, National Defense Medical College, Tokorozawa, Saitama 359-8513, Japan; 6 Department of Biosciences and Informatics, School of Fundamental Science and Technology, Keio University, Yokohama, Kanagawa 223-8522, Japan

**Keywords:** CD34-positive hematopoietic cells (CD34 cells), cell proliferation, cytokines

## Abstract

Low-molecular-weight heparin/protamine microparticles (LH/P MPs) serve as carriers for controlled release of heparin-binding cytokines. LH/P MPs were stably coated onto plastic surfaces by drying. The purpose of this study is to evaluate a culture method for selective expansion of CD34+ cells using LH/P MPs as cytokine-binding matrix. Ficoll-purified mouse bone marrow cells (mouse FP-BMCs) containing CD34+ cells were cultured on LH/P MP-coated plates in the presence of stem cell factor (SCF), thrombopoietin (Tpo), and Flt-3 ligand (Flt-3) in hematopoietic progenitor growth medium (HPGM) supplemented with 4% heat-inactivated fetal bovine serum (FBS). After 8 days of culture, the total cell count increased 4.6-fold, and flow cytometry analyses revealed that 23.8% of the initial cells and 57.4% of the expanded cells were CD34 positive. Therefore, CD34+ cells were estimated to have increased 11.0-fold. In contrast, cultured CD34+ cells on uncoated tissue culture plates increased 5.8-fold in an identical medium.

## Introduction

Cellular expression of the CD34 antigen identifies a morphologically and immunologically heterogeneous cell population that is functionally characterized by its *in vitro* capability to generate clonal aggregates derived from early and late progenitors in hematopoiesis and its *in vivo* capacity to reconstitute the myeloid hematopoietic system in a supralethally irradiated host.^1^ On the other hand, there are several recent reports that a certain CD34+ cells form cooperative vascular networks as endothelial progenitor cells,^2^ and human CD34+ cells have been used in clinical trials for treatment of myocardial infarction.^3^ Furthermore, synergistic actions of CD34+ hematopoietic cells and mesenchymal stem/progenitor cells in vascularizing bioengineered tissues have been reported.^4^ Thus, CD34+ cells are important cells for vascularizing bioengineered tissues as well as transplantation of bone marrow. Bone marrow and peripheral blood are the sources of immature hematopoietic precursors identified as CD34+ cells. Large numbers of CD34+ cells can be obtained from massive marrow harvests, such as for transplantation purposes.^1,5,6^ A diagnostic marrow sample contains only a small number of CD34+ cells, while even fewer are present in the peripheral samples taken under steady-state conditions. Recently developed cell separation procedures such as panning, combination of galactose-bound vinyl polymer and soybean agglutinin,^7^ and immunomagnetic beads allow for the recovery of a highly enriched CD34+ cell population.^8^ However, this is generally achieved through a specific and often unacceptable cell loss. In contrast, *in vitro* expansion of CD34+ cells may be of particular relevance for cell transplantation.

CD34+ cells undergo regulated proliferation, conservation, and differentiation in the bone marrow microenvironment. Therefore, the cell pool can be preserved while allowing controlled cell proliferation and differentiation.^9,10^ CD34+ cells are localized in stem cell niches and local area networks in the microenvironment, where they interact with the components of their niche, including stromal cells, extracellular matrix proteins, heparan sulfate proteoglycans, and cytokines. Regional variation in these components within the hematopoietic microenvironment may create niches that are specific to cells at various stages of differentiation.^11–13^ However, the identity and structural characteristics of macromolecules that mediate the formation of these niches are not well known. In addition, a method for selecting *ex vivo* amplification of CD34+ cells from bone marrow has not yet been established.^14^

Human CD34+ cells proliferate and mature in semisolid media when stimulated with exogenous hematopoietic cytokines such as stem cell factor (SCF), thrombopoietin (Tpo), and Flt-3 ligand (Flt-3).^15–18^ They also proliferate in association with bone marrow–derived stromal cells,^19,20^ although biologically active amounts of hematopoietic cytokines cannot be detected in stromal culture supernatants.^18^ It is possible that hematopoietic cytokines are synthesized by stromal cells but remain bound to them and/or their extracellular matrix. In fact, it has been demonstrated that natural and recombinant hematopoietic cytokines, such as IL-3 and granulocyte macrophage colony–stimulating factor (GM-CSF), can be absorbed by heparan sulfate, the major sulfated glycosaminoglycan in bone marrow stroma.^17,18^ Furthermore, SCF, Tpo, and Flt-3 bind to LH/P MP-coated plates. These cytokines, once bound, can be presented in their biologically active form to the CD34+ cells.^19,20^ Other studies have demonstrated that heparinoids (heparin, heparan sulfate, and other heparin-like molecules) serve as cofactors to promote binding of SCF to high-affinity receptors, thus enhancing its activity.^19,20^ These findings may have important implications for the use of heparinoids as biomaterials for selective *ex vivo* expansion of CD34+ cells.

Because heparin and low-molecular-weight heparin (LH) are known to interact with a variety of functional proteins, such as growth factors, cytokines, extracellular matrix components, and adhesion molecules,^21,22^ heparin could be used as a therapeutic agent for various pathological conditions in which these functional proteins are involved.^23^ However, high-dose heparin cannot be used because of the excessive risk of bleeding.^24^ In contrast, LH has pharmacological and practical advantages as well as low protein binding, which produces a low, stable, and predictable anticoagulant response, thereby obviating the need for laboratory monitoring for dose adjustment.^25^. In addition, one or two subcutaneous injections per day are sufficient to maintain therapeutic concentrations because of the long plasma half-life of LH.^25^

On the other hand, protamine, a purified mixture of proteins obtained from fish sperms, neutralizes heparin and LH by forming a stable complex, which lacks anticoagulant activity.^26^ Protamine is also used clinically as an antidote to reverse the anticoagulant activity of heparin following cardiopulmonary bypass and in cases of heparin-induced bleeding.^27^ In this study, we used LH (for comparison with heparin) as a heparinoid, and protamine to prepare LH/P MPs.^28^ The purpose of this study is to evaluate LH/P MPs as a stem cell niche for the controlled release of hematopoietic cytokines, which stimulate the selective growth of CD34+ cells. We report here that CD34+ cells derived from mouse bone marrow exhibited a significantly higher proliferation on LH/P MP-coated plates in hematopoietic progenitor growth medium (HPGM) supplemented with adequate cytokines and 4% fetal bovine serum (FBS) than those on uncoated plates

## Materials and methods

### Preparation of LH/P MPs

LH/P MPs were synthesized as described previously.^28^ Briefly, 0.35 ml of protamine solution (10 mg/ml; Mochida Pharmaceutical Co., Tokyo, Japan) was added drop by drop to 0.7 ml of LH (fragmin solution: 6.4 mg/ml; Kissei Pharmaceutical Co., Tokyo, Japan) with vigorous vortexing for approximately 2 min. To maximize the production of microparticles, protamine and LH were mixed in a ratio of 1:2 (vol:vol) in this study. The LH/P MPs solution (1.05 ml) was then washed twice with phosphate-buffered saline (PBS) to remove nonreactants, and finally adjusted to 1 ml with PBS. More than 6 mg of dry LH/P MPs was obtained from 1.05 ml of the LH/P MPs solution.

### Binding of LH/P MPs onto plastic plates and preparation of LH/P MP-coated plates

**Figure 1.**
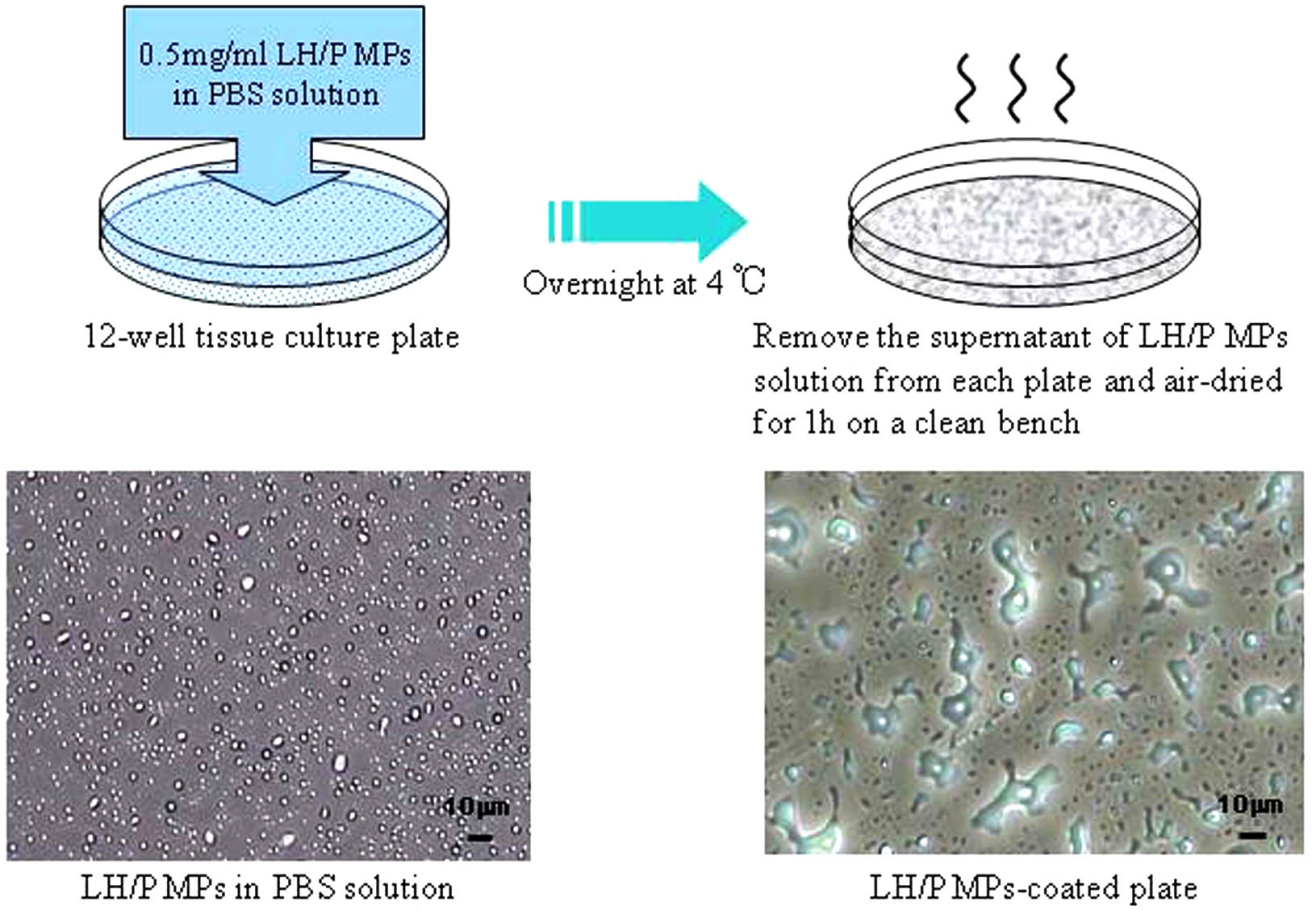
Preparation of LH/P MP-coated plates. LH aqueous (6.4 mg/ml) and protamine solutions (10 mg/ml) were mixed in a ratio of 1:2 (vol:vol) to produce LH/P MPs. The LH/P MPs consist of insoluble round complexes of approximately 1.0–0.5 μm in diameter (average: approximately 0.7 μm in diameter) and can be stably coated onto plastic surfaces by drying.

Twelve-well culture plates (well area: 360 mm^2^; Sumitomo Bakelite Corp., Tokyo, Japan) were coated overnight at 4°C with 1 ml of 0.5 mg/ml LH/P MPs in the PBS solution. LH/P MPs solutions were removed from the wells by pipetting. LH/P MPs were loosely bound to the plastic surface, and the unbound particles were easily washed away with PBS. The LH/P MPs were stably coated onto the plastic surface by air-drying the 12-well plate-bound LH/P MPs for 1 h on a clean bench (Figure 1).

### Isolation of mouse FP-BMCs from femurs of mice

Bone marrow cells were isolated by flushing both femurs of a C57BL/6J mouse (Japan SLC, Inc., Tokyo, Japan), and debris were eliminated by filtration through a 100 μm cell strainer (BD Biosciences Discovery Labware, Bedford, MA, USA). Furthermore, red blood cells and platelets were eliminated by density gradient centrifugation with Ficoll-Paque (1.077 g/ml; Amersham Biosciences, Uppsala, Sweden) at 400 *g* for 30 min. Mouse FP-BMCs were suspended in Dulbecco’s modiﬁed Eagle’s medium (DMEM) without FBS before culturing. These animal experiments were approved and performed in accordance with the guidelines for animal experimentation of the National Defense Medical College (Saitama, Japan).

### Culture of FP-BMCs from mouse bone marrow

Prepared mouse FP-BMCs were plated at an initial density of about 100,000 cells/well on either 12-well tissue culture plates (Sumitomo Bakelite Corp., Tokyo, Japan) or LH/P MP-coated plates in 2.5 ml of HPGM (Lonza Japan Corp., Tokyo, Japan) supplemented with the indicated concentrations of inactivated FBS (JRH Biosciences, Inc., KS, USA) containing antibiotics (100 U/ml penicillin G and 100 μg/ml streptomycin), and the indicated concentrations of recombinant human SCF (Acris Corp., Hiddenhausen, Germany), Tpo (Acris Corp.), and Flt-3 (Acris Corp.). The cells were cultured at 37°C in an atmosphere of 5% CO_2_ and 100% relative humidity for the indicated periods. Almost all mouse FP-BMCs were easily suspended in the medium by pipetting the cell cultures, and the number of cells was counted using a hemocytometer (Sigma-Aldrich, Tokyo, Japan). The culture medium was not changed during the 8-day culture.

### Selection of culture medium

Mouse FP-BMCs were plated on LH/P MP-coated plates using either one of the four types of basal media (Asahi Techno Glass Co., Tokyo, Japan) including DMEM, (RPMI-1640), DMEM/Ham’s nutrient mixture F12 (DMEM/F12), and HPGM. Each medium was supplemented with 4% FBS, antibiotics (100 U/ml penicillin G and 100 μg/ml streptomycin), and 1/2× cytokines (SCF (10 ng/ml), Tpo (20 ng/ml), and Flt-3 (20 ng/ml)). The concentrations of SCF (20 ng/ml), Tpo (40 ng/ml) and Flt-3 (40 ng/ml) in HPGM are represented as 1×.

### Flow cytometry analyses

Cultured mouse FP-BMCs (approximately 1.0 × 10^5^ cells) were suspended in 1 ml of stain buffer (FBS (10 ml) + PBS (40 ml) + NaN_3_ (0.5 g)) and centrifuged twice at 3000 rpm for 2 min to wash the cells. A 5-µl aliquot of Phycoerythrin PE-labeled anti-mouse CD34 (BD Biosciences Pharmingen, San Diego, CA, USA) was added to the pellet and incubated in an ice bath at 4°C for 15 min. The cells were washed twice with 1 ml of stain buffer, and then 500 µl of IsoFlow (Beckman Coulter, Fullerton, CA, USA) was added to the pellet. The samples were analyzed using EPICS^®^XL (Beckman Coulter).

### Statistical analyses

Statistical analyses were performed by Student’s *t*-test. Probability values were calculated using the software, Stat Mate III for Windows (ATMS Co., Tokyo, Japan). The values of *p* < 0.05 were considered to be statistically significant.

## Results

### LH/P MP-coating and determination of culture conditions for expansion of CD34+ cells

Although added LH/P MPs were loosely bound to the plastic surface, particles were easily washed away from the surface with PBS. Alternatively, LH/P MPs were stably coated onto the plastic surface by air-drying bound LH/P MPs for 1 h on a clean bench (Figure 1). While most FP-BMCs did not bind to both the LH/P MP-coated and uncoated tissue culture plates, only a small number of cells (<20%) loosely bound to both the LH/P MP-coated and uncoated tissue culture plates. Almost all of these bound cells could be easily released by washing with the culture medium.

**Figure 2.**
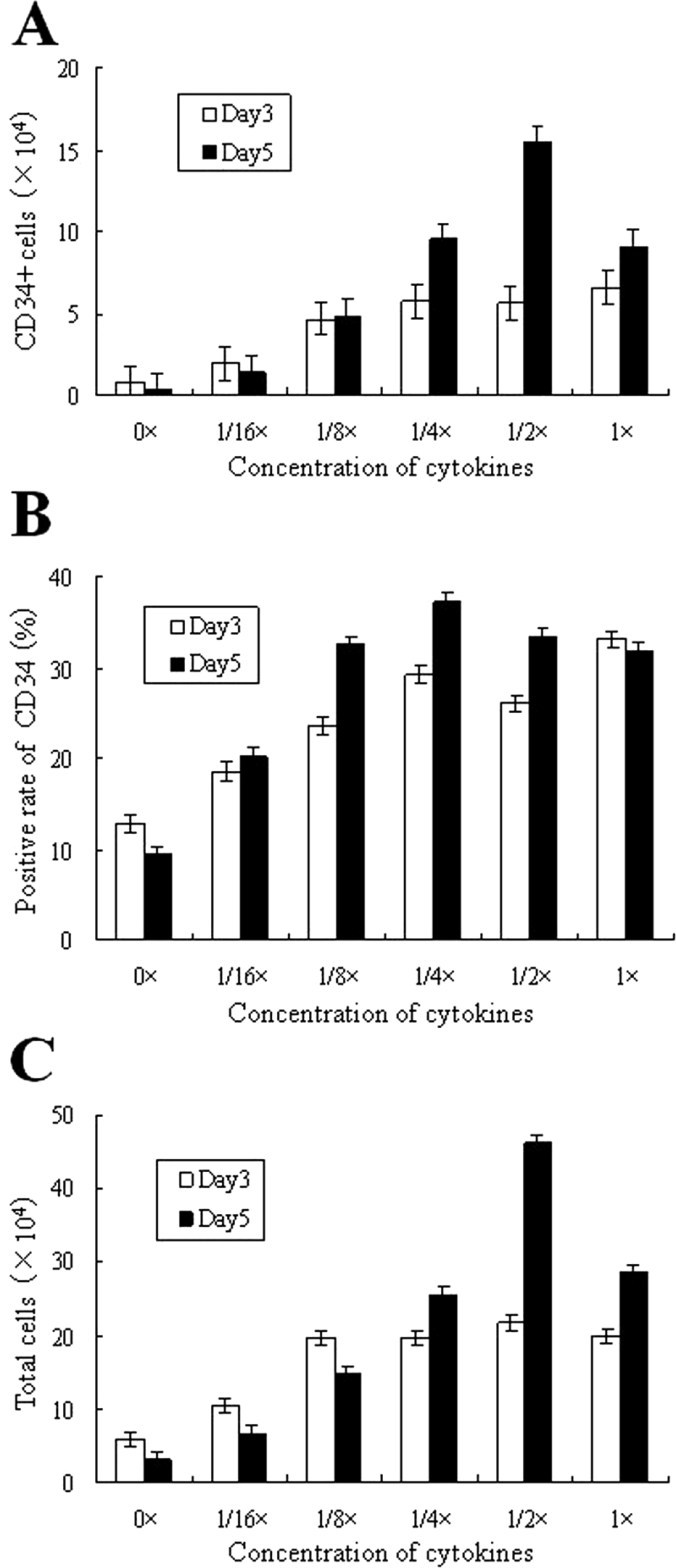
Optimal concentrations of cytokines. Optimal concentrations of SCF/Tpo/Flt-3 were determined on LH/P MP-coated plates incubated for 3 and 5 days in the presence of the indicated cytokines in HPGM supplemented with 4% FBS. The concentrations of SCF (20 ng/ml), Tpo (40 ng/ml), and Flt-3 (40 ng/ml) in HPGM are represented as 1×. White bars (Day 3) and black bars (Day 5) represent total cells (mouse FP-BMCs) (C) and CD34+ cells (A), which were calculated using data from flow cytometry analyses shown in (B). The ratio of CD34+ to total cells is represented in (B). Data represent mean ± SD of six determinations.

**Figure 3.**
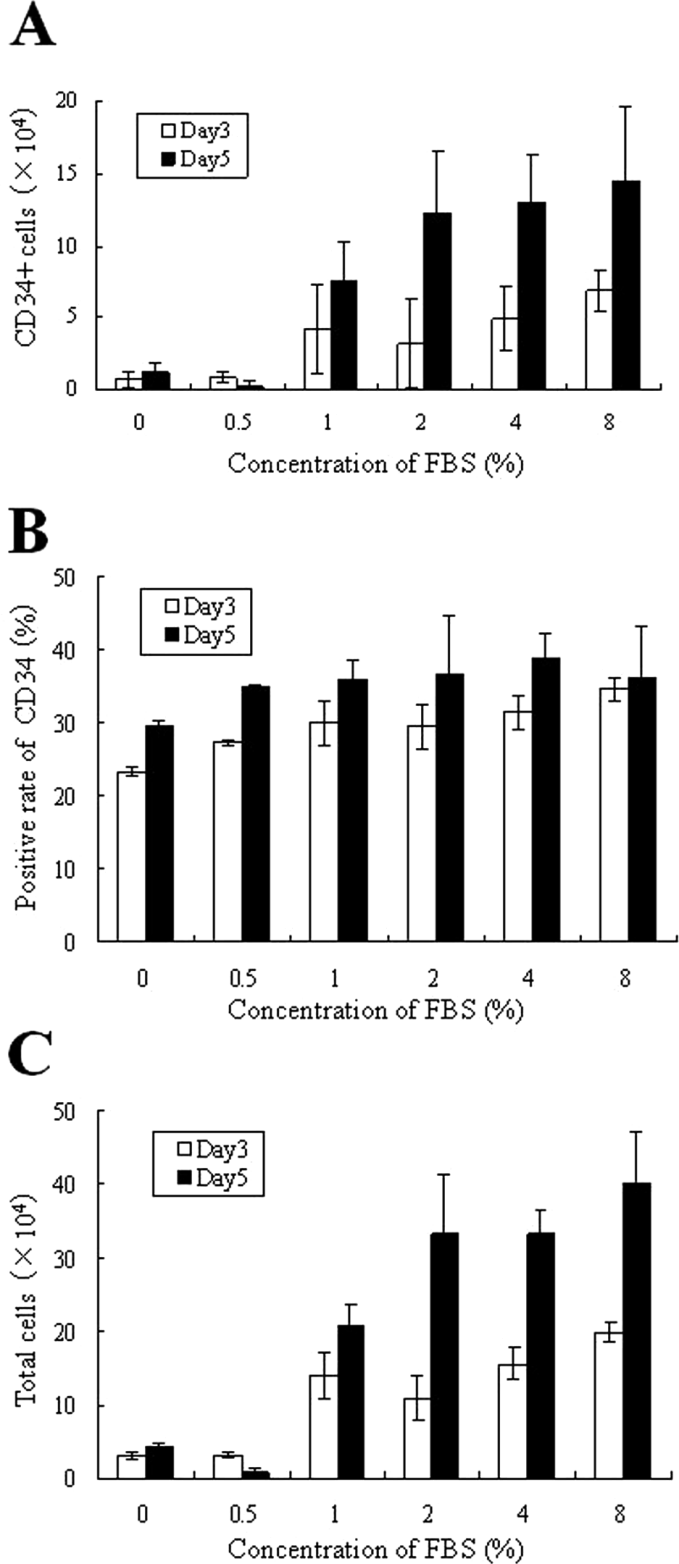
Optimal concentrations of serum. Optimal concentrations of FBS were determined on LH/P MP-coated plates incubated for 3 and 5 days in the presence of the indicated concentrations of FBS with 1/2× cytokines in HPGM. White bars (Day 3) and black bars (Day 5) represent total cells (mouse FP-BMCs) (C) and CD34+ cells (A), which were calculated using data from flow cytometry analyses shown in (B). The ratio of CD34+ to total cells is represented in (B). Data represent mean ± SD of six determinations.

The optimal concentrations of SCF, Tpo, Flt-3, and FBS in HPGM were tested for maximum expansion of mouse FP-BMCs and CD34+ cells on LH/P MP-coated plates (Figures 2 and 3). In this study, we used 12-well culture plates using 2.5 ml of the medium for culturing mouse FP-BMCs because over 50,000 cells were required to evaluate the marker CD34 by flow cytometry as well as for estimating the cell numbers. When mouse FP-BMCs (approximately 100,000 cells) were plated on 12-well tissue culture plates, few cells adhered to the plates, and no cell colonies were obtained in these cultures. However, the optimal expansion of CD34+ cells as well as total cells (mouse FP-BMCs) was obtained on LH/P MP-coated tissue culture plates containing concentrations (1/2×) of SCF (10 ng/ml), Tpo (20 ng/ml), and Flt-3 (20 ng/ml) in HPGM supplemented with 4% FBS (Figure 2). The total cell number increased 4.6-fold under these conditions after 5 days of culture (Figure 2C). Because flow cytometry analysis (Figure 2B) revealed that 23.8% of original mouse FP-BMCs and 33.4% of the expanded cells after 5 days of culture were CD34+, CD34+ cells were estimated to have increased 6.5-fold after 5 days of culture (Figure 2A).

Since FBS was required for expansion of cells, the optimal concentration of FBS was also determined in the presence of the cytokines (1/2×), SCF (10 ng/ml), Tpo (20 ng/ml), and Flt-3 (20 ng/ml) in HPGM (Figure 3). The total cell (mouse FP-BMCs) number increased 3.3-fold by 4% or more FBS after 5 days of culture (Figure 3C). Since flow cytometry analysis (Figure 3B) revealed that 23.8% of original mouse FP-BMCs and 39.0% of the expanded cells after 5 days of culture were CD34+, CD34+ cells were estimated to have expanded 5.5-fold after 5 days of culture (Figure 3A). Considering mouse FP-BMC growth and the ratio of CD34+ cells to the total cells, we chose concentrations (1/2×) of SCF (10 ng/ml), Tpo (20 ng/ml), and Flt-3 (20 ng/ml) in HPGM supplemented with 4% FBS for optimal culture conditions for CD34+ cells on LH/P MP-coated plates.

### Evaluation of various culture media for growth of CD34+ cells

**Figure 4.**
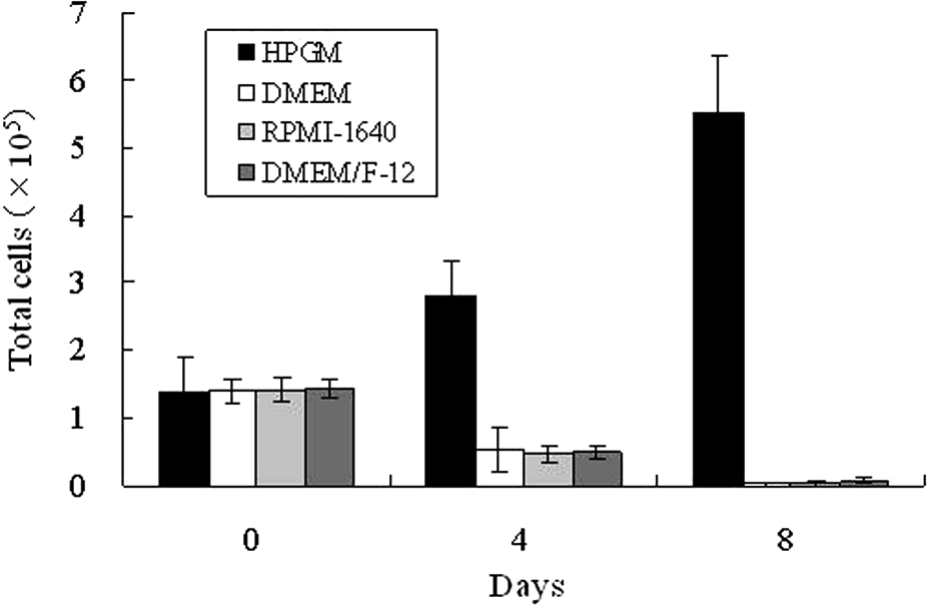
Evaluation of various culture media for growth of CD34+ cells. To define the optimal media for expansion of CD34+ cells, mouse FP-BMCs were plated on LH/P MP-coated tissue culture plates containing various basal culture media supplemented with 4% FBS, antibiotics, and 1/2× cytokines. Data represent mean ± SD of six determinations.

To define the optimal media for expansion of CD34+ cells, mouse FP-BMCs were plated on LH/P MP-coated tissue culture plates containing various basal culture media, including DMEM, RPMI-1640, DMEM/F12, and HPGM supplemented with 4% FBS, antibiotics, and 1/2× cytokines (SCF (10 ng/ml), Tpo (20 ng/ml), and Flt-3 (20 ng/ml)). The CD34+ cells as well as mouse FP-BMCs were able to grow only in HPGM (Figure 4).

### Assessment of expansion of CD34+ cells

**Figure 5.**
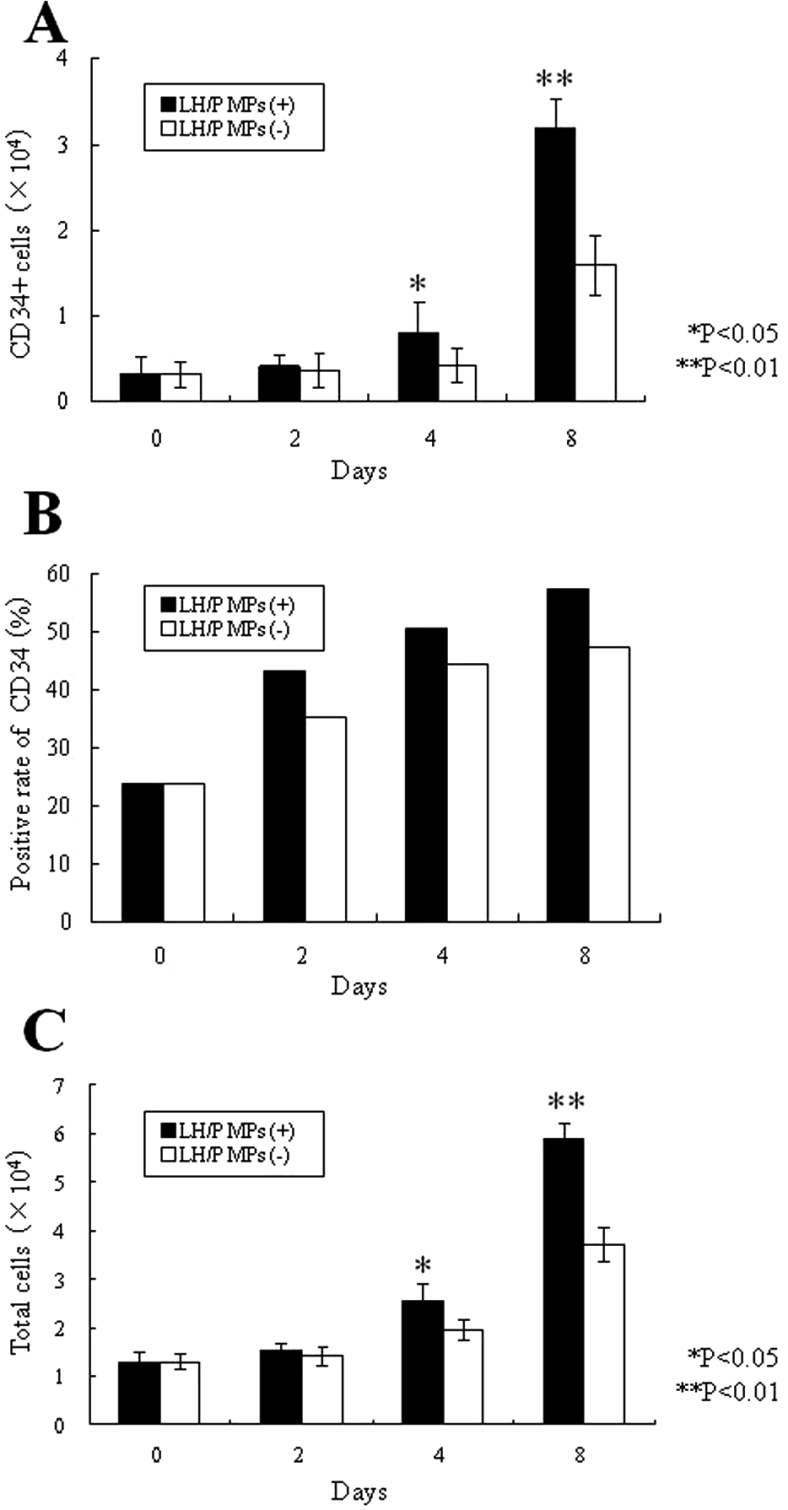
Expansion of CD34+ cells on LH/P MP-coated plates. Expansion of total cells (mouse FP-BMCs) and CD34+ cells on both LH/P MP-coated and uncoated tissue culture plates was examined in HPGM supplemented with 1/2× cytokines and 4% FBS for 8 days. Total cells (C) were counted using a hemocytometer, and CD34+ cells (A) were calculated using flow cytometry data (B) to determine the ratio of CD34+ cells to total cells (mouse FP-BMCs). Data represent mean ± SD of six determinations.

When mouse FP-BMCs were plated on both LH/P MP-coated and uncoated tissue culture plates, the cells did not adhere to either of the plates. However, these cells were able to grow in suspension culture with concentrations (1/2×) of SCF (10 ng/ml), Tpo (20 ng/ml), and Flt-3 (20 ng/ml) in HPGM supplemented with 4% FBS. The total cell number increased by 4.6- and 2.9-fold after 8 days of culture on LH/P MP-coated and uncoated tissue culture plates, respectively (Figure 5C). Since flow cytometry analysis revealed that 23.8% of initial mouse FP-BMCs, and 57.4% and 47.5% of the expanded cells on LH/P MP-coated and uncoated tissue culture plates, respectively, were CD34+ (Figure 5B), CD34+ cells were estimated to have expanded 11.0-fold after 8 days of culture on LH/P MP-coated plates (Figure 5A). The ratios of CD34+ cells to total cells on LH/P MP-coated plates were always higher than those on uncoated plates as determined on Days 2, 4, and 8.

## Discussion

We previously reported that LH/P MPs, which are approximately 1 μm in diameter, facilitate the fibroblast growth factor-2 (FGF-2) injection.^28^ FGF-2-containing LH/P MPs induce substantial vascularization and fibrous tissue formation because of the gradual release of FGF-2 molecules from the LH/P MPs. In this study, LH/P MP-coating as a stem cell niche stimulated the growth of CD34+ cells in HPGM with adequate cytokines (SCF, Tpo, and Flt-3). Mouse FP-BMCs in the presence of concentrations (1/2×) of cytokines (SCF (10 ng/ml), Tpo (20 ng/ml), and Flt-3 (20 ng/ml)) in HPGM supplemented with 4% FBS exhibited 4.6- and 2.4-fold expansion after 8 days of culture on LH/P MP-coated and uncoated tissue culture plates, respectively. Furthermore, since flow cytometry analysis (Figure 5B) revealed that the percentage of CD34+ in the expanding cells on LH/P MP-coated plates increased from 23.8% to 57.4% during the 8 days of culture (Figure 5B), the CD34+ cells expanded 11.0- and 5.8-fold after 8 days of culture on LH/P MP-coated and uncoated tissue culture plates, respectively. Thus, the LH/P MP-coated plate in the presence of appropriate cytokines is a useful culture method for selective expansion of CD34+ cells in mouse FP-BMCs. However, we still need to characterize the expanded cell population with respect to various surface antigens in addition to CD34 and different morphologies/functions in the expanded cells.

CD34+ cells are localized in stem cell niches and local area networks in the microenvironment of bone marrow, where they interact with components of their niche including stromal cell surfaces, extracellular matrix proteins, heparan sulfate proteoglycans, and cytokines.^17,18^ Heparan sulfate proteoglycans are found in the extracellular matrix produced by stromal cells. They are prime candidates for selectively retaining CD34+ cells stimulatory factors in the niche (stromal layers), and they may regulate hematopoiesis.^19^ In addition, the immobilization of cytokines (SCF, Tpo, and Flt-3) onto LH/P MP-coated plates may provide a matrix-like stem cell niche that is bioactive for the proliferation of CD34+ cells.

It is recognized in polymer chemistry that positively and negatively charged polymers interact ionically.^29^ Basic protamine molecules complexed with acidic molecules such as LH form microparticles through ionic interactions. The amounts of LH and protamine on the LH/P MP-coated plates used for culturing CD34+ cells did not alter for at least 8 days (data not shown). However, it probably appears that polypeptides such as SCF, Tpo, and Flt-3, once bound to the LH/P MPs are gradually released from the LH/P MP-coated plates and decreased by half within 5 days of culture.^19^ Although LH/P MPs are not biodegradable *in vitro*, the inclusion of various cytokines, such as SCF, Tpo, and Flt-3, into the coating will provide an excellent cytokine-containing matrix for CD34+ cells.^15–18^ We have previously demonstrated that SCF, Tpo, and Flt-3 are heparin-binding proteins, that all three interact with LH/P MPs, and that the immobilized cytokines are gradually released into the culture medium over 6 days.^19,20^ Furthermore, it has been shown that heparin addition enhances the expansion of cord blood hematopoietic progenitor cells in three-dimensional coculture with stromal cells.^30^

The induction of hematopoietic cell differentiations stimulated by several late-acting cytokines may be considered as consequence of proliferation of earlier hematopoietic population such as CD34+ cells stimulated by several cytokines such as SCF and leukemia inhibitory factor (LIF).^31^ The majority of late-acting cytokines such as CSFs, interleukins (ILs), Tpo, and erythropoietin (EPO) support the proliferation and maturation of lineage-committed progenitors and the functional properties of differentiated cells. Intermediate-acting, lineage-nonspecific cytokines including IL-3, IL-4, and GM-CSF are mainly directed toward progenitor cells in the intermediate stages of hematopoietic development.^32^ Therefore, it may be possible that a specific hematopoietic population in BMCs can be selectively expanded by various combinations of appropriate cytokines using the LH/P MP-coated plates.

Dalteparin is an LH that has an anticoagulant activity much lower than that of the native heparin and can therefore be administered subcutaneously.^25,26^ Therefore, dalteparin as LH was used to prepare LH/P MPs in these studies. No bleeding complications were observed in animals injected with LH/P MPs in a previous study.^28^ In addition, since the two components for LH/P MPs, LH, and protamine, are already in clinical use, their clinical safeties are ensured.
